# Opportunities and challenges associated with fecal progesterone metabolite analysis

**DOI:** 10.14202/vetworld.2018.1466-1472

**Published:** 2018-10-20

**Authors:** Innocent Damudu Peter, Abd Wahid Haron, Faez Firdaus Abdullah Jesse, Mokrish Ajat, Mark Hiew Wen Han, Wan Nor Fitri, Muhammad Sanusi Yahaya, Mohammed Saad M. Alamaary

**Affiliations:** 1 Department of Veterinary Clinical Studies, Faculty of Veterinary Medicine, Universiti Putra Malaysia, Serdang, Selangor Darul Ehsan, Malaysia; 2 Department of Theriogenology, Faculty of Veterinary Medicine, University of Maiduguri, Maiduguri, Nigeria; 3 Institute of Tropical Agriculture and Food Security, Universiti Putra Malaysia, Serdang, Selangor Darul Ehsan, Malaysia; 4 Department of Veterinary Pre Clinical Science, Faculty of Veterinary Medicine, Universiti Putra Malaysia, Serdang, Selangor Darul Ehsan, Malaysia

**Keywords:** non-invasive methods, progesterone metabolite, progesterone, reproductive cycles

## Abstract

Conventionally, plasma or milk progesterone evaluations are used to determine the reproductive status of female animals. Collection of such samples is often associated with difficulties of animal handling and restraint. Measurable quantities of progesterone metabolites are found in feces of animals. Their concentrations are known to be well correlated to plasma progesterone levels and are, therefore, used as non-invasive samples for assessing reproductive function in a wide range of animal species. Although the analysis of fecal progesterone metabolites has been widely accepted in many laboratories, several factors are known to affect the results from this valuable analytical technique. Some of these factors include storage/transportation media for fecal samples, type of solvent that is used for extraction of progesterone metabolites from feces, and the type and sensitivity of an assaying technique employed. Although fecal progesterone metabolites analysis is associated with some difficulties, it can effectively be used to monitor reproductive function in a wide range of animal species. This review aims to highlight the usefulness of fecal progesterone metabolite analysis as a non-invasive technique in monitoring reproductive function in animals. The article mainly focuses on the many opportunities and challenges associated with this analytical technique.

## Introduction

**Figure-1:**
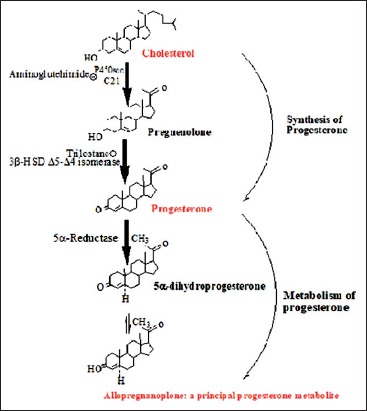
Conversion of cholesterol to progesterone and metablolism of progesterone into one of its principle metabolite, alporegnanolone (Illustration made using chemdraw, perkinelmwr informatics).

In mammals, native progesterone is mainly synthesized by the ovary, the adrenal gland, and placenta [1,2]. Progesterone regulates the estrous cycle and is the principal hormone responsible for the maintenance of pregnancy in female animals [1]. Native progesterone is synthesized from cholesterol after its conversion to pregnenolone by cytochrome P450scc, a protein located in the inner surface of the inner mitochondrial membrane. Pregnenolone is further converted into progesterone in a reaction that is catalyzed by 3β-hydroxysteroid dehydrogenase (Figure-1) [3]. Progesterone is metabolized by the liver into several metabolites and are thereafter excreted in feces (Figure-1) [4-8]. There are at least 18 progesterone metabolites (2 pregnanediones, 8 mono-hydroxylated pregnanes, and 8 di-hydroxylated pregnanes), each having a unique chemical structure and polarity [5,9-11]. Excretion time of progesterone metabolites has been found to take a fairly long time in non-ruminants (approximately 48 h) than in ruminants (12-24 h) [12-15].

A better understanding of fundamental reproductive processes in animals is dependent on the collection of blood samples and analysis of reproductive hormones therein. However, in most animal species, the collection of blood samples is accompanied with stress and difficulties of animal handling and restraint, and in some cases, there may be a need for expertise [9,16-19]. This is even more difficult especially in aquatic and some free-living terrestrial animals [2,20,21]. Furthermore, the stress of blood sample collections could increase the risks of abortion and or death of the dam, especially in early pregnancies in some species of animals [22,23]. These issues have led to the development of non-invasive techniques to study reproductive processes using fecal samples. This methodology has gained considerable importance due to the ease of sample collection and analysis [18,20-22,24]. Although sedatives and tranquilizers are indicated for restraint in free-ranging and intractable animals, chemical restraint methods are known to pose significant health risks and could alter plasma progesterone concentrations [25-27]. Other non-invasive samples such as urine and milk samples can be used for such purpose in animals, the simplicity of obtaining fecal samples offers a superior advantage [28,29]. Milk samples are usually obtained from lactating animals while urine samples will require the fixing of catheters and some animal restraint. Fecal samples, however, can be collected at any time with ease and without any stress or restraint [23,24,30,31].

The development of non-invasive techniques to study reproductive and endocrinological processes in animals was primarily developed to circumvent the difficulties and stress associated with blood sample collection. Non-invasive techniques offer opportunities and can be applied in a wide range of animal species. However, there are challenges associated with such non-invasive methods. These involve problems ranging from choice of appropriate transport and storage media for fecal samples to sensitivity and specificity of a chosen assay technique employed in non-invasive technologies. This review aims to discuss the opportunities and challenges associated with the study of fecal progesterone metabolite as a non-invasive tool in the assessment of reproductive function in animals.

### Progesterone Metabolites Deposits in Sample Matrices

There is quite a lot of literature on studies relating to steroid hormones and reproduction in cattle using samples such as blood, milk, fecal, or hair samples [32]. These matrices have unique characteristics and also have advantages and disadvantages. Blood samples remain the optimum sample for determination of reproductive function in animals as this sample provides the true concentration of circulating steroid hormones at any given time [12]. Collection of blood, milk, and hair samples requires animal restraint. Restraint in animals for blood sample collection is already known to be a difficult and stressful procedure. Milk sample collection is limited to lactating animals and collection will also require restraint. To determine short-term steroid levels in animals, blood, milk, and feces are preferred samples [32]. Hair samples are better suited for the determination of long term steroid hormones levels as hair is not affected by the pulsatile release of the hormone into bloodstream [33].This allows hormones to accumulate in hair throughout its growth, making it possible to assess long-term gonadal activity without the need for serial and continuous sample analysis in animals [34]. So far, some studies have reported having used hair progesterone concentrations for determining reproductive function in animals [32,35]. However, such studies revealed weak correlations between hair progesterone concentrations and ovarian activity. This, therefore, necessitates the need for in-depth investigations to elucidate on the mechanism of steroid deposition in hair and validation of the analysis of hair steroid hormone concentrations before its reliable application in applied research in animals.

### Fecal Progesterone Metabolite Analysis Technologies

Immunological techniques such as radioimmunoassay (RIA) or enzyme immunoassay designed for progesterone relies on specific or broad-spectrum antibodies and have been frequently used to measure progesterone and its immunoreactive metabolites in fecal samples [6,7,10,17,18,27,36]. Using either assay, it is possible to characterize ovarian cycles [16], screen open cows [10], and assess reproductive function in wildlife [8,36-40]. The choice of which immunological technique to employ is dependent on factors such as assay technique involved, type of information required, route of excretion of the metabolites, as well as the practicability of sample collection [18,19].

Other analytical methods such as high-performance liquid chromatography (HPLC) have been developed and are frequently used in several laboratories for quantifying progesterone metabolites in biological samples [7,19,41,42]. HPLC separates progesterone metabolites according to their respective properties [9,10,36,43]. Separation is easily carried out on a reverse phase HPLC stationary phase and an isocratic solvent system using acetonitrile and distilled water at different proportions [25]. Gas chromatography-mass spectrometry (MS), a variant of HPLC, has equally been employed in the analysis of progesterone metabolites. This technique has been shown to provide very accurate results in terms of concentrations of progesterone metabolite in different sample matrices [36].

### Application of fecal steroid analysis

Several studies have demonstrated that plasma progesterone concentrations and fecal progesterone metabolites are correlated [9,13,30,44]. Based on this similarity, progesterone metabolite evaluations have been used to study the reproductive physiology of many animals [21]. Some indicators of reproductive function in animals have been successfully determined using fecal progesterone evaluations [5,8,16,40]. Non-invasive methodologies allow for long-term monitoring of endocrine parameters without necessarily causing undue disturbance to the animal under investigation [27,45]. Results of progesterone metabolite evaluations that are used in assessment in fecal samples from several animals species are presented in Table-1 [2,7,8,14-17,20,21,25,39,40,42,44,46-70]. Fecal progesterone metabolite analysis has so far been applied in several species of animals, namely whales [8], deer [16,45], cattle [25], goats [30], sheep [46], felids [71], rhinoceros [72], hamsters [47], elephant [48], gazelle [49] and baboons [73], and hippopotamus [50].

**Table-1:** Application of fecal progesterone metabolite analysis in assessing reproductive function in animals.

Reproductive parameter	Species	Major metabolite	Type of assay	Reference
Ovarian function	Cows	5a-pregnan-3a-ol-20-one	RIA/HPLC	Rabiee *et al*. [7], Yimer *et al*. [25],
estrus cycle	Deer	-	EIA	Masunda *et al*. [42],
	Anteater	-	EIA	Desaulniers *et al*.[44]
	Elephant	5a-P-3OH	EIA	Pereira *et al*. [16], Polegato *et al*.[52]
	Sheep	Pregnanediol-3-glucuronide	EIA	Knott *et al*.[53]
	Gazelle		EIA	Ghosal *et al*. [20], Illera *et al*. [21],
	Hamsters	-	RIA	Ghosal *et al*. [48],
	Rhinoceros	-	EIA	Thitaram *et al*.,[54]
	Rhinoceros	-	RIA	Čebulj-Kadunc *et al*.[46]
	Whale	Pregnanediol-3-glucuronide	HPLC	Mohammed *et al*. [49],
	Jaguars		EIA	Wojtusik *et al*.[55]
	Panda	-	EIA	Chelini *et al*.[47]
	Monkeys	-	RIA	Schwarzenberger *et al*. [39],
	Sea otters	-	EIA	Van der Goot *et al*.[56]
	Warthogs	-	EIA	Rolland *et al*.[8]
	Dholes	-	RIA/EIA	Conforti *et al*.[57]
	Sloth	-	EIA/HPLC	Budithi *et al*.[58]
	Mink	pregnanediol-glucuronide	EIA	Silvestre *et al*.[59]
	Numbat	-	EIA	Larson *et al*.[40]
	Armadillos	-	EIA	Berger *et al*.[60]
	Hippopotamus	-	EIA	Khonmee *et al*.[61]
	Aoudad	pregnanediol-3-glucuronide	EIA	Troll *et al*.[17]
	Takin		EIA	Nagl *et al*.[47]
	Python	-	HPLC	Hogan *et al*.[62]
	Pronghorn	-		Howell-Stephens *et al*.[63]
		-		Flacke *et al*.[50]
		-		Abáigar *et al*.[14]
				Adkin *et al*.[15]
				Bertocchi *et al*. [64], Curry *et al*.[69]
				Kersey *et al*. [65]
Pregnancy	Leopard cat	5α-pregnan-3α-ol-20-one	HPLC	Hogan *et al*. [62], Adachi *et al*.[66]
	Cows	5α-pregnan-3α-ol-20-one	EIA	Howell-Stephens *et al*. [63],
	Deer	-	EIA	Isobe *et al*.[67]
	Gazelle	-	EIA	Pereira *et al*. [16], Knott *et al*. [53],
	Panda	-	EIA	Krepschi *et al*.[68]
	Zebra	-	EIA	Mohammed *et al*. [49],
	Dugongs	-	EIA	Wojtusik *et al*.[55]
	Deer	5α-pregnan-3α-ol-20-one	EIA/HPLC	Bertocchi *et al*. [64], Curry *et al*.[69]
				Kersey *et al*.[65], Ncube *et al*. [65,70]
				Burgess *et al*.[2]
				Mithileshwari *et al*. [51]
Anestrus	Gazelle		EIA	Van der Goot *et al*.[56]
	Aoudad		EIA	Abáigar *et al*. [14]

EIA=Enzyme immunoassasy, RIA=Radioimmunoassasy, HPLC=High-performance liquid chromatography

### Challenges in the Analysis of Fecal Progesterone Metabolite Analysis

Although the use of fecal samples offers ease of sampling as compared to blood collection, there are a number of challenges that are associated with using this sample matrix for analysis.

### Transport and storage of fecal samples

Most times, it takes a fairly long time for samples to arrive at the laboratory for analysis. Failure to properly preserve fecal samples allows gut bacteria and their enzymes to further metabolize progesterone metabolites therein [19,74-76]. At present, there is no consensus on the best method of preserving fecal samples. Fecal samples from cows have been reported to have a significant decrease in total progesterone metabolite levels over short- and medium-term storage without preservatives [42,72].

To obtain accurate results from the analysis of fecal progesterone metabolite analysis, samples ought to be analyzed soon after excretion [77,78]. The concentration of progesterone metabolites in feces decreases significantly when left at ambient environmental conditions without preservation [42]. Transport and preservation of fecal samples are best achieved by storage in methanol or ethanol before analysis. Such medium ensures stabilization of progesterone metabolites for several weeks [72,79,80]. Both chemicals have bacteriostatic properties and can also inactivate several bacterial enzymes [78,81]. Ethanol is a good preservative for samples relating to such studies [75], but the practicability of using this preservative under field conditions is quite challenging. Most often, sampling is done at remote locations and transportation from sampling site to the laboratory with such hazardous compounds poses some health hazard. Furthermore, samples that contain more than 24% alcohol are considered explosive and also classified as flammable which will require more care while handling [75].

Alternatively, fecal samples can be dried, or an on-field extraction can be performed or even carry out a solid phase extraction. These methods are considered to be alternative methods for processing fecal samples after collection for later use [72]. Drying of fecal samples is reported to be able to stabilize progesterone metabolites for up to 180 days [72].

Freezing fecal samples soon after collection is another valuable method of preserving progesterone metabolites in fecal sample [72,82]. Unfortunately, freezing of fecal samples is not always possible as in sometimes, sampling is done in remote locations where the habitat of free-ranging animals may be far away from sources of electricity necessary for refrigeration [72,83]. Even when such facilities are available in the field, there is the tendency that freezers and refrigerators quickly fill up with bulky fecal materials.

### Extraction protocol

The extraction of progesterone metabolites from the fecal sample for determination of reproductive function is a common procedure in several laboratories. Despite the widespread use of such a technique, the comparison of results between laboratories is difficult due to the variation in extraction methods and choice of analytical technique adopted by different laboratories [11,25,43,84,85]. Furthermore, the differences observed in metabolism and excretory pattern of steroid hormones in different species and breeds of animals makes extrapolation of results much more difficult and sometimes misleading [18]. Commonly employed extraction procedure for progesterone metabolites includes vortexing a known weight of dried or wet fecal sample suspended in an extraction solvent usually ethanol or methanol [5,7,10]. To get maximum recovery of progesterone metabolites from fecal samples, certain laboratories increase the number of extraction steps. This procedure is also believed to eliminate the effects of disturbing elements from the fecal matrix. Most often, two or more solvents are used in the extraction of progesterone metabolites of known polarities from feces. Polar metabolites are preferably extracted in distilled water while non-polar metabolites are extracted in hexane and ether [86].

### Specificity of assay systems used in the analysis of fecal progesterone metabolites

Due to their widespread availability and their all-encompassing nature, progesterone and immunoassays are continuously used to quantify progesterone metabolites in feces [5]. Native progesterone, however, is not found in feces or only present in minute amounts [51]. Antibodies used in the development of such kits are very specific for progesterone, and they also cross-react with progesterone metabolites in feces. Such cross-reactivity is mainly due to structural similarities between progesterone and its several metabolites. The use of such kits for progesterone metabolite evaluation in feces is, therefore, less suitable. Results obtained from diagnostic kits manufactured with antibodies to progesterone only give concentrations of total immunoreactive metabolites in the sample matrix [5]. Progesterone metabolites are generally categorized as 5α or 5β pregnanes based on the presence of either a 20-oxo, or a 20α, or rather a 20β -OH group [5]. Enzyme immunoassays that are developed with group-specific antibodies would, therefore, provide more accurate and reliable results [51].

Results obtained from LC-MS and HPLC are more accurate and serve as valid alternatives to the ambiguous nature of results provided by immunoassay systems. LC techniques offer higher specificity over immunoassay systems and can be used in quantifying individual metabolite. However, limitations do exist in terms of cost and availability. In addition, requisite expertise is often required for LC techniques [85].

### Other factors

Other lesser factors affecting the analysis of progesterone metabolites in feces are feeding and feed intake, as well as factors affecting the metabolism of progesterone. Rabiee [7] and Hutchinson *et al*. [87] both opined that feed and dry matter intake affect the total concentration of progesterone metabolites in feces of cows. Furthermore, feed restricted cows have been shown to have a higher concentration of progesterone metabolites in feces [7]. In other studies, it was shown that excretion rates of progesterone metabolites were found to be affected by the total weight of excreted feces [6]. Metabolism of progesterone has also been shown to be affected by the percentage of dry matter intake as well as other diet composition.

## Conclusion

Although measurements of plasma progesterone remain the ideal choice for deterring reproductive function in animals, fecal progesterone metabolite evaluations can be conveniently used as an alternative method for the same purpose. Collection of fecal samples is quite easy, and multiple samples can be collected over time. However, factors such as the degradative activity of fecal bacteria on progesterone metabolites, transportation conditions, storage, and extraction method, as well as sensitivity and specificity of assays affect results. This challenge can be addressed with the use of appropriate preservation and extraction methods as well as utilizing sensitive and specific assay systems for analyzing progesterone metabolite concentration in feces.

## Author’s Contributions

AWH, IDP, and FFAJ conceived the idea and designed the main frame of this manuscript as part of IDP’s research work under the supervision of AWH. IDP made the first draft and was read and corrected by AWH, FFJA, MA, and MHWH. IDP wrote the second draft, which WNF, MSY, and MSMA critically read and revised the manuscript for intellectual content All authors read and approved the final manuscript.
